# Atypical cartilaginous tumor imaging findings in the distal phalanx of the left thumb: case report and literature review

**DOI:** 10.3389/fonc.2025.1407012

**Published:** 2025-02-24

**Authors:** Hong Wang, Xinyi Tang, Yuting Wang, Xiaoyan Peng, Yujia Yang

**Affiliations:** Department of Medical Ultrasound, West China Hospital of Sichuan University, Chengdu, China

**Keywords:** atypical cartilaginous tumor, chondrosarcoma, hands and feet, imaging, diagnosis

## Abstract

**Background:**

Atypical cartilaginous tumors (ACTs) usually occur in long bones rather than in the hands or feet. To date, detailed imaging features of ACTs in the hands or feet were reported in only a few case reports.

**Case presentation:**

We report a case of an Asian woman in her early 80s who presented to our hospital with a painless mass in the distal phalanx of the left thumb. Radiography and computed tomography (CT) showed osteolytic destruction and cortex thickening in the distal phalanx with extension into soft tissue. Magnetic resonance imaging (MRI) demonstrated a local soft tissue signal mass with isosignal intensity in T1 and heterogeneous high-signal intensity in T2. Color Doppler ultrasound suggested that the tumor was hypovascularized. The patient underwent amputation, and histological analysis revealed an ACT. The patient’s symptoms improved postoperatively, with no recurrence as of the 3-year follow-up.

**Conclusion:**

Meanwhile, this study also reviewed the comparable diagnostic methods of ACT and chondrosarcoma. The analysis of previous similar cases showed that preoperative imaging diagnosis of ACT can be challenging and multimodal imaging appears to be beneficial in diagnosing ACTs and malignant chondrosarcoma grade II/III in the hands and feet.

## Introduction

In the 5th Edition of the World Health Organization (WHO) tumor classification of bone and soft tissue tumors, published in 2020, atypical cartilaginous tumor/chondrosarcoma grade I (intermediate-locally aggressive) (ACT/CS1) in the 2013 version was divided into two diseases: ACT and CS1. The former was retained in the classification of intermediate-locally aggressive, and the latter was upgraded to malignancy. The new version of the guideline differentiates between ACT and CS1 based on the location of the tumor: tumors located within the appendicular bone both in long and short tubular bones are called ACT, while those occurring in the axial skeleton (pelvis, scapula, and skull base flat bones) are called CS1 to reflect the poorer clinical outcome of these tumors at these sites. The most common sites affected by ACT are the femur, humerus, and tibia. ACTs of the hands and feet are not common. Because of the rarity of ACT in the hands and feet, the imaging literature on ACT is scarce and mainly comprises older or small case series, as well as case reports describing conventional radiographic features (1, 2). Preoperative imaging diagnosis of ACT can be challenging. We report a case of ACT in the distal phalanx of the left hand thumb and made a detailed report on its imaging performance. The imaging features of CS and ACT in hands and feet were summarized and reviewed.

## Case report

**Figure 1:**
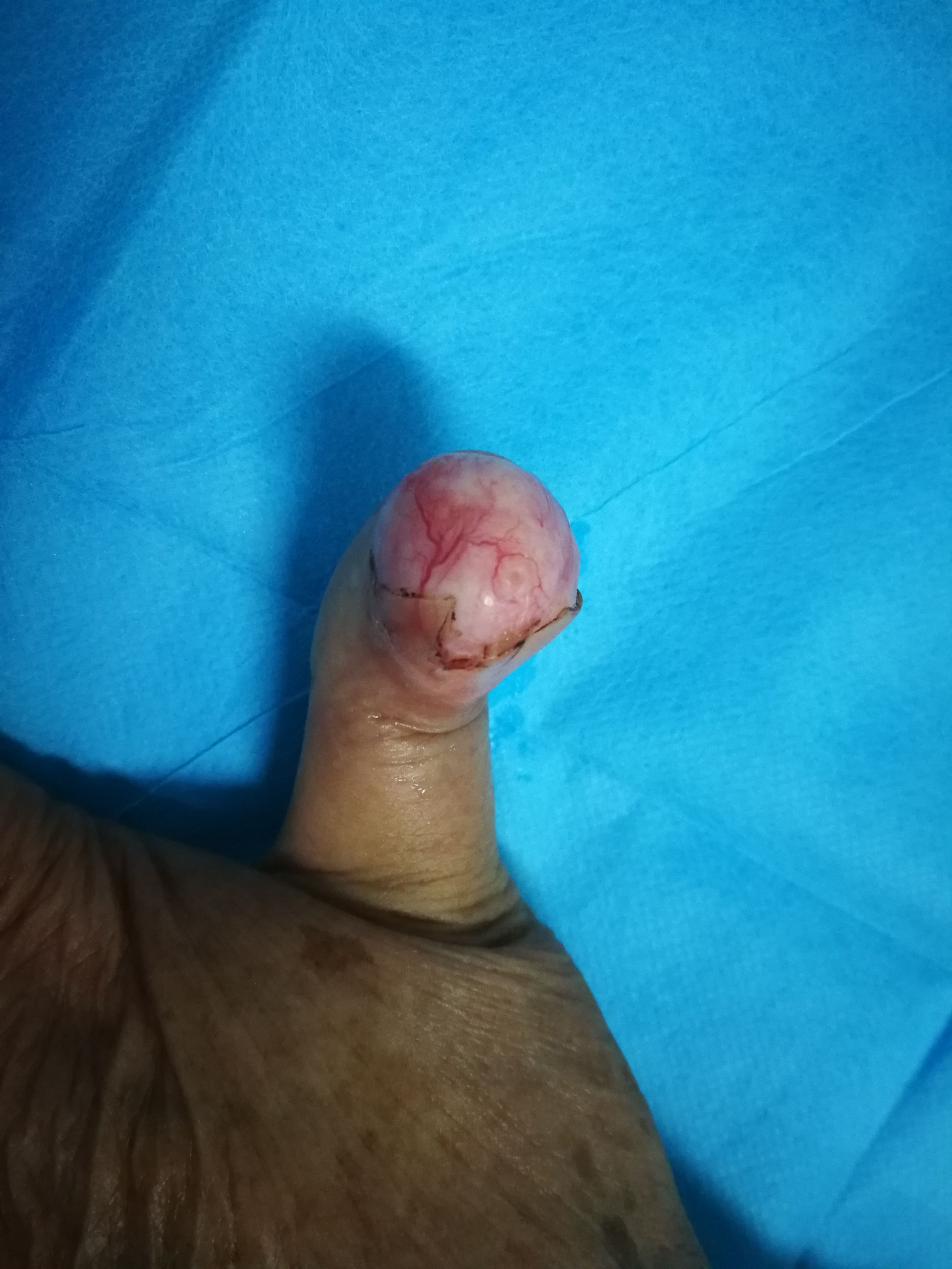
Preoperative photographs of the patient.

**Figure 2:**
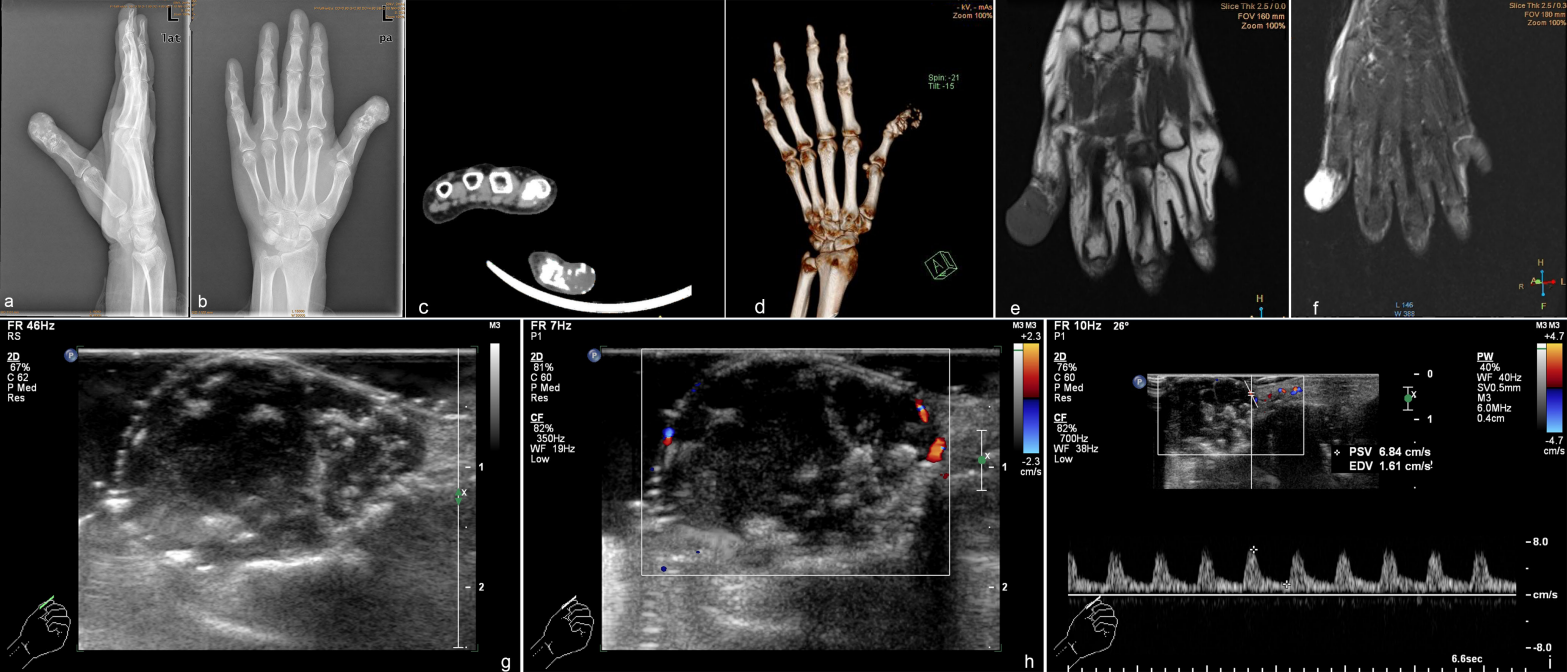
Preoperative appearance of the patient’s hand shows a destructive cortex and osteolytic change with soft tissue masses. **(A)** Lateral radiograph; **(B)** radiograph. Computed tomography imaging **(C)** and 3D view **(D)** of atypical cartilaginous tumor of the hand shows osteolytic destruction of the distal phalanx of the thumb. Magnetic resonance imaging. **(E)** T1-weighted imaging shows a low-signal shadow of the mass; **(F)** T2-weighted imaging shows a heterogeneously high signal intensity. Ultrasound demonstrated a hypoechoic mass with calcification **(G)** and a dotted blood flow signal **(H)**. **(I)** Spectrum Doppler shows the arterial spectrum.

The authors have read the case report (CARE) Checklist (2016), and the manuscript was prepared and revised according to the CARE Checklist (2016). An Asian woman in her early 80s presented with a palpable and painless mass in the distal phalanx of the left thumb. The patient noticed that mass 2 years ago, and the mass swelled progressively in the 2 years and its growth accelerated in the past 6 months, which prompted the woman to go to the hospital for medical assessment. Upon physical examination, the patient had a swollen left hand thumb, giving it a drumstick appearance (**Figure 1**). The patient claimed a history of type 2 diabetes and hypothyroidism with no known trauma or any masses elsewhere. Our patient did not have Ollier’s disease, Maffucci syndrome, or hereditary exostosis and had no evidence of prior enchondroma. The results of routine blood tests and biochemical tests were normal. To further clarify the diagnosis, the patient received medical imaging examinations. The radiograph showed a mass in the distal phalanx of the left thumb with osteolytic bone destruction (**Figures 2A, B**). In computed tomography (CT) images, osteolytic destruction of the distal phalanx of the thumb with a local soft tissue density mass was observed (**Figures 2C, D**). Magnetic resonance imaging (MRI) also showed cortical destruction of the distal phalanx of the thumb and a local soft tissue signal mass of approximately 20 mm × 19 mm × 26 mm with isosignal intensity in the T1-weighted image (T1WI) and heterogeneously high signal intensity in the T2-weighted image (T2WI) (**Figures 2E, F**). The mass invaded the interphalangeal joint. In addition, the patient underwent ultrasound examination. Ultrasound imaging demonstrated a hypoechoic mass (25 mm × 18 mm × 22 mm) with patchy calcification in the distal phalanx of the left thumb (**Figures 2G–I**). The mass had a clear margin and irregular shape. The bone cortex around the mass is discontinuous and not smooth. Color Doppler showed a dot blood flow signal within the mass. Subsequent chest radiography and bone scans did not reveal any metastatic lesions.

**Figure 3:**
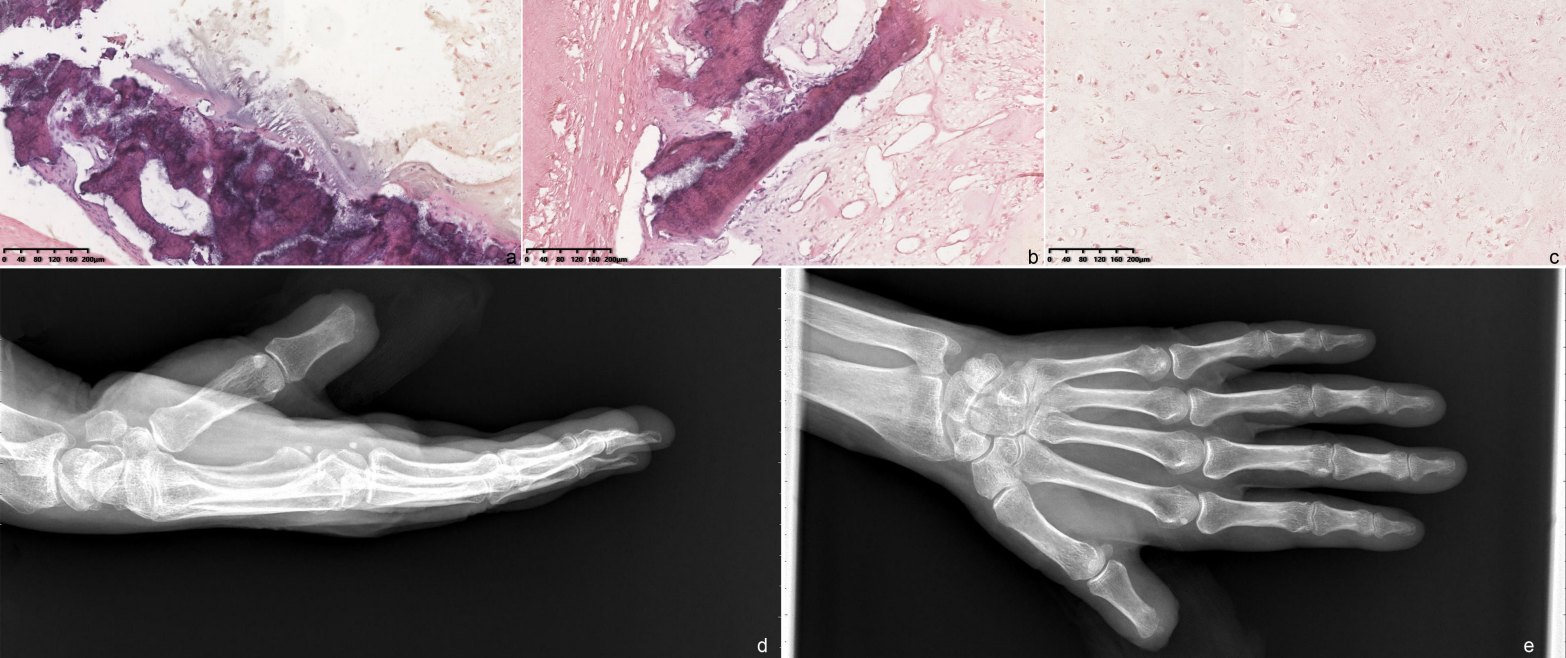
Hematoxylin and eosin-stained section of the tumor (×10 magnification). **(A)** Microscopically, there are many slightly heterotopic chondrocytes with obvious heteromorphism and a caryocinesia phase. **(B, C)** Most of the tumor cells show invasive growth, accompanied by a few bone trabeculae. Postoperative image of the patient. **(D)** Lateral radiograph; **(E)** radiograph.

With the above imaging findings, the patient received amputation of the left thumb distal phalanx to achieve radical excision of the mass. The lesion tissues were stained with hematoxylin-eosin (HE). Microscopically, blue-stained cartilaginous matrix was found, and chondrocytes showed obvious heteromorphism. The caryocinesia was observed, with invasive growth accompanied by a few bone trabeculae. Pathologic findings are consistent with a chondrogenic tumor, showing intermediate-locally aggressive behavior. Combined with tumor location (appendicular skeleton, not the axial skeleton), imaging findings (a mass with osteolytic bone destruction), and an aggressive clinical behavior (rapid growth in the last 6 months), the tumor was diagnosed as an ACT (**Figures 3A–C**). The postoperative x-ray examination showed a good surgical outcome (**Figures 3D, E**). After the surgery, the patient experienced an improvement in her symptoms and reported a better quality of life. During the 3-year follow-up, there was no evidence of recurrence detected by MRI or CT scans. Informed consent was obtained from the subject described in this report.

## Discussion

Although the 2013 version proposed the concept of ACT, it did not provide specific diagnostic criteria, and it was difficult to apply to clinicopathological diagnosis. The 2020 version clarified the diagnostic criteria for ACT: tumors occurring in the appendicular bones (long and short tubular bones) are called ACT, while those occurring in the axial bones (flat bones including the pelvis, scapula, and skull base) are called CS1. ACT and CS1 have the same histological characteristics, but the prognosis of appendicular bone tumors is significantly better in axial bone (3). The new version of the guidelines divides ACT/CS1 into ACT and CS1 based on the anatomical location of the tumor, with the former retained as intermediate and the latter upgraded to malignant disease. ACT involving bones of the hands and feet is uncommon. Early diagnosis is helpful for the treatment and prognosis of the disease. Radiography, CT, and MRI are helpful for tumor biopsy and treatment.

In order to differentiate ACT and CS imaging characteristics of the hands or feet, we searched the following three databases: PubMed, Embase, and Cochrane Library, according to the Preferred Reporting Items for Systematic Reviews and Meta-Analyses guidelines (4). We also manually searched additional relevant studies using the references of the systematic reviews that were published previously. All of the searches were performed from inception to 22 August 2022, and imposed restrictions on the English language. The inclusion criteria specified studies and case reports describing patients with primary CS or ACT confirmed by pathologically involving the hand or the feet who received image examination.

The following search strategy was used for PubMed and was modified to suit Embase and Cochrane Library databases.

#1 (chondrosarcoma [Title/Abstract]) OR (atypical cartilaginous tumor[Title/Abstract])

#2 (small bone [Title/Abstract] OR hand [Title/Abstract] OR foot [Title/Abstract])

#1 AND #2

Next, we screened the titles and abstracts of these articles, excluding articles in which the lesion did not involve the hands or feet, those not describing the imaging examination features, and those in which the tumors were secondary. Periosteal chondrosarcoma, clear cell chondrosarcoma, mesenchymal chondrosarcoma, and dedifferentiated chondrosarcoma were excluded because they have different biological behaviors (5). **Supplementary Figure 1** illustrates the study selection process.

As a result, 32 articles (6–37) discussing 92 cases were selected. The characteristics of the selected cases, including the present case, are listed in **Supplementary Table 1**. All included cases were primary chondrosarcoma confirmed by pathology. There were 49 male and 43 female patients. All patients were adults, except for a 12-year-old male patient reported by Gupta et al. (28). Among all patients, 90 patients underwent radiographic examination, 8 patients underwent CT, 17 patients underwent MRI, 6 patients underwent CE-MRI, and 16 patients underwent bone scans. In **Supplementary Table 1**, the radiography and CT imaging manifestations of chondrosarcoma and ACT in hands and feet were mainly seen as cortical destruction, soft tissue mass, and osteolytic change, which characteristically contained calcification. Additional features that may be present included endosteal scalloping and periosteal reaction. On MRI, a total of 9 cases provided detailed information on T1WI and T2WI. These lesions appeared as hyposignal or isosignal on T1WI images. On T2WI images, the above lesions were hypersignal compared with skeletal muscle. CE-MRI showed an enhanced area among all patients who underwent CE-MRI examination. In addition, Tos et al. (23) reported a case showing a rounded high-intensity image on coronal STIR and a low-intensity heterogeneous lesion on sagittal SET1. The bone scanning results of 16 patients all showed abnormal radioactive accumulation, one of which indicated that the tumor was hypervascular. Only the lesion in this case report was examined by ultrasound. Ultrasound imaging demonstrated cortical destruction and a hypoechoic mass with patchy calcification. Color Doppler showed that the tumor was hypovascularized. Resection, amputation, and ray resection were commonly used in treatment.

Considering that in the previous classification versions, ACT and CS1 were classified into the same category (ACT/CS1), but in the new classification version published in 2020, the intermediate-locally aggressive chondrosarcoma grade I (CS1) occurring in the hand or foot is uniformly classified into ACT, in the following analysis of imaging findings, we excluded the data without clear pathological classification in **Supplementary Table 1**. The data in **Supplementary Table 1** were reclassified as two categories: intermediate-locally aggressive ACT and high-malignant chondrosarcoma grade II/III (CS2/3), for comparison of imaging findings. After the reclassification, 10 cases of ACT and 35 cases of CS2/3 were retained for further analysis. Of the 10 cases of ACT, it is more common in male than in female patients (female:male = 3:7). The age range was 29–87 years, with an average age of 52 years. In 35 cases of CS2/3, it is more common in female than in male patients (female:male = 20:15). The age range was 30–85 years, with a mean age of 63 years old. In terms of symptoms, the two categories may show pain, swelling, or both. In ACT, 20% of patients presented with pain (2/10), 20% also presented with swelling (2/10), and 30% had both (3/10). In CS2/3, 43% patients presented with pain (15/35), 17% presented with swelling (6/35), and 40% had both (14/35). Imaging findings showed that in ACT, 80% (8/10) of patients showed cortical destruction, 40% (4/10) displayed calcification, 70% (7/10) showed soft tissue mass, 40% (4/10) showed osteolytic destruction, and only 1 case (10%) showed periosteal reaction. In CS2/3, 83% (29/35) of patients showed cortical destruction, 80% (28/35) showed calcification, 74% (26/35) showed soft tissue mass, osteolytic destruction was observed in 11% (4/35), and periosteal reaction was observed in 2 cases (6%). In summary, both ACT and CS2/3 are common in middle-aged and elderly patients. ACT is more common in men, while CS2/3 is more common in women. In imaging findings, both of the two categories showed cortical destruction and soft tissue mass. Calcification was more likely to occur in malignant CS2/3. Therefore, there are certain difficulties in the imaging of the two categories, and pathological examination is still needed to distinguish them.

In clinical practice, radiography and CT remain the mainstay for the initial detection of chondrosarcoma or ACT of small bones and are helpful for characterization of the lesion (38). The main application of MRI is the preoperative assessment for staging the extent of disease (1, 2). However, there is a paucity of data on the use of PET/MRI in ACT or CS of small bones. However, some scholars (39) have noted that PET/MRI can provide additional functional information to supplement the morphologic mapping and histopathology of these tumors. It is expected that future research will highlight a potential role for PET/MRI in the management of CS or ACT of small bones. Ultrasound examination is noninvasive, painless, and economical. It can not only show small lesions of the bone cortex and observe the structural relationship between the tumor and surrounding blood vessels but also show different degrees of bone destruction, periosteal reaction, and soft tissue invasion (40). Color Doppler flow imaging can also provide hemodynamic information. These results also suggest that multimodal imaging is helpful to improve the diagnostic efficiency of CS or ACT.

This study also has some limitations. First, since only hands and feet with imaging findings were collected, the epidemiological data collected in this study are not comprehensive. Second, because the collected literature in **Supplementary Table 1** were published before the release of the new version in 2020, we failed to classify ACT and CS of the imaging information in some articles. In addition, for the analysis of imaging findings, fewer cases were included in this study, and no statistical analysis was made on the differences. The differential diagnosis of imaging features between ACT and malignant CS2/3 needs further study.

In conclusion, although ACT is very rare in hands and feet, the possibility of ACT should also be considered for soft tissue tumor occurring in middle-aged and elderly men whose clinical manifestations are mainly pain and swelling, and the imaging findings are cortical destruction, soft tissue mass, and osteolytic change. Multimodal imaging may be helpful to improve the diagnostic efficiency of ACT.

## Data Availability

The datasets presented in this study can be found in online repositories. The names of the repository/repositories and accession number(s) can be found in the article/**Supplementary Material**.

## Supplementary material

Supplementary Figure 1 A flowchart showing the literature search and analysis process.

Supplementary Table 1 List of primary atypical cartilaginous tumor or chondrosarcoma cases receiving imaging examination. LF, left foot; RH, right hand; PP, proximal phalanx; PIP, proximal interphalangeal point; LH, left hand; MP, metacarpophalangeal; Gd, gadolinium-enhanced; MC, metacarpal.

## References

[1] Deckers C Steyvers MJ Hannink G Schreuder HWB de Rooy JWJ Van Der Geest ICM. Can MRI differentiate between atypical cartilaginous tumors and high-grade chondrosarcoma? A systematic review. Acta Orthop. (2020) 91:471–8. doi: 10.1080/17453674.2020.1763717 PMC8023913 32429792

[2] Alhumaid SM Alharbi4th A Aljubair H. Magnetic resonance imaging role in the differentiation between atypical cartilaginous tumors and high-grade chondrosarcoma: an updated systematic review. Cureus. (2020) 12:e11237. doi: 10.7759/cureus.11237 33269165 PMC7704161

[3] Wells ME Childs BR Eckhoff MD Rajani R Potter BK Polfer EM. Atypical cartilaginous tumors: trends in management. J Am Acad Orthop Surg Glob Res Rev. (2021) 5:e21.00277. doi: 10.5435/JAAOSGlobal-D-21-00277 PMC8683228 34913887

[4] Moher D Liberati A Tetzlaff J Altman DG PRISMA Group. Preferred reporting items for systematic reviews and meta-analyses: the PRISMA statement. PloS Med. (2009) 6:e1000097. doi: 10.1371/journal.pmed.1000097 19621072 PMC2707599

[5] Bindiganavile S Han I Yun JY Kim HS. Long-term outcome of chondrosarcoma: a single institutional experience. Cancer Res Treat. (2015) 47:897–903. doi: 10.4143/crt.2014.135 25687868 PMC4614192

[6] Lewis MM Marcove RC Bullough PG. Chondrosarcoma of the foot. A case report and review of the literature. Cancer. (1975) 36:586–9. doi: 10.1002/1097-0142(197508)36:2<586::AID-CNCR2820360239>3.0.CO;2-9 1157020

[7] Hernàndez-Vaquero D Cima-Suarez M Garcia-Pravia C. Chondrosarcoma of the bones of the hand. Rep two cases Arch Orthop Trauma Surg. (1991) 110:265–8. doi: 10.1007/BF00572885 1931370

[8] Zhou LB Zhang HC Dong ZG Wang CC. Chondrosarcoma of the toe: A case report and literature review. World J Clin Cases. (2022) 10:9132–41. doi: 10.12998/wjcc.v10.i25.9132 PMC9477028 36157642

[9] Jones HB Murphree J Suryavanshi JR Osemwengie BO Rosqvist S Cox CT. Multifocal chondrosarcoma of the hand: Case report and review of the literature. Clin Case Rep. (2021) 9:e04352. doi: 10.1002/ccr3.4352 34136252 PMC8190542

[10] Vasilakaki T Tsavari A Skafida E Koulia K Myoteri D Grammatoglou X. Chondrosarcoma of the proximal phalanx of the fourth digit: A rare location. Case Rep Oncol. (2012) 5:566–9. doi: 10.1159/000343915 PMC3506041 23185160

[11] Hamada K Tomita Y Ueda T Yoshikawa H. Chondrosarcoma of the hand: radiologic evaluation at early stage. Eur J Orthop Surg Traumatol. (2010) 20:233–5. doi: 10.1007/s00590-009-0544-5

[12] Miyake A Morioka H Yabe H Anazawa U Morii T Miura K. A case of metacarpal chondrosarcoma of the thumb. Arch Orthop Trauma Surg. (2006) 126:406–10. doi: 10.1007/s00402-006-0134-5 16557368

[13] Hatori M Watanabe M Kotake H Kokubun S. Chondrosarcoma of the ring finger: A case report and review of the literature. Tohoku J Exp Med. (2006) 208:275–81. doi: 10.1620/tjem.208.275 16498237

[14] Ollero P Vanden Dungen S Cermak K Kinnen L. Metacarpal chondrosarcoma, from negligence to rareness: a case report and review of the literature. Acta Orthop Belg. (2021) 87:541–3. doi: 10.52628/aob 34808730

[15] Knapp P Aviles A Najarian C. Low-grade chondrosarcoma of the proximal phalanx:A rare presentation. Case Rep Orthop. (2019) 2019:6402979. doi: 10.1155/2019/6402979 31001441 PMC6437730

[16] Özmanevra R Calikoglu E Mocan G Erler K. Grade 2 chondrosarcoma of the great toe: an unusual location. Am Podiatr Med Assoc. (2019) 109:393–6. doi: 10.7547/18-097 31599673

[17] García-Jiménez A Chanes-Puiggrós C Trullols-Tarragó L Pulido-García MC. Chondrosarcoma of the hand bones: A report of 6 cases and review of the literature. J Handb Surg Asian Pac Vol. (2019) 24:45–9. doi: 10.1142/S2424835519500085 30760143

[18] Pop DL Motoc AGM Hărăguş HG Ciupe BC Iacob M Vermeşan D. Conventional chondrosarcoma in the right hand with the invasion of the pisiform and the hamate bones - case report. Rom J Morphol Embryol. (2017) 58:271–5. 28523331

[19] Hills AJ Tay S Gateley D. Chondrosarcoma of the head of the fifth metacarpal treated with an iliac crest bone graft and concurrent Swanson’s arthroplasty. J Plast Reconstr Aesthet Surg. (2014) 67:e84–7. doi: 10.1016/j.bjps.2013.10.023 24183060

[20] De Moraes FB Linhares ND De Souza Domingues PM Warzocha VN Soares JM. Calcaneal chondrosarcoma: a case report. Rev Bras Ortop. (2014) 49:409–13. doi: 10.1016/j.rboe.2014.04.020 PMC4511569 26229837

[21] Mondal SK. Chondrosarcoma of the distal phalanx of the right great toe: Report of a rare Malignancy and review of literature. J Cancer Res Ther. (2012) 8:123–5. doi: 10.4103/0973-1482.95191 22531530

[22] Decomas A Lurie D Meyer M. Chondrosarcoma of the foot. Am J Orthop (Belle Mead NJ). (2011) 40:37–9. 21720585

[23] Tos P Artiaco S Linari A Battiston B. Chondrosarcoma in the distal phalanx of index finger: Clinical report and literature review. Chir Main. (2009) 28:265–9. doi: 10.1016/j.main.2009.02.002 19345602

[24] Toriyama K Kamei Y Yagi S Uchibori M Nishida Y Torii S. Reconstruction of the first and second metatarsals with free vascularised double-barrelled fibular graft after resection of a chondrosarcoma. J Plast Reconstr Aesthet Surg. (2009) 62:e580–3. doi: 10.1016/j.bjps.2008.11.062 19136322

[25] Hatori M Watanabe M Kokubun S. Chondrosarcoma of the distal phalanx of the great toe. J Am Podiatr Med Assoc. (2007) 97:156–9. doi: 10.7547/0970156 17369324

[26] Mohammadianpanah M Torabinejad S Bagheri M Omidvari S Mosalaei A Ahmadloo N. Primary chondrosarcoma of phalanx. Foot. (2004) 14:159–63. doi: 10.1016/j.foot.2004.01.001

[27] Masuda T Otuka T Yonezawa M Kamiyama F Shibata Y Tada T. Chondrosarcoma of the distal phalanx of the second toe: a case report. J Foot Ankle Surg. (2004) 43:110–2. doi: 10.1053/j.jfas.2004.01.002 15057858

[28] Gupta K Radhika S Vasishta RK. Chondrosarcoma of calcanaeum in a 12-year-old male patient: A case report. Diagn Cytopathol. (2004) 31:399–401. doi: 10.1002/dc.20130 15540179

[29] Patil S De Silva MV Crossan J Reid R. Chondrosarcoma of small bones of the hand. J Handb Surg Br. (2003) 28:602–8. doi: 10.1016/S0266-7681(03)00149-9 14599838

[30] Lo EP Pollak R Harvey CK. Chondrosarcoma of the foot. J Am Podiatr Med Assoc. (2000) 90:203–7. doi: 10.7547/87507315-90-4-203 10800275

[31] Ralph BG Barrett J Kenyhercz C Cuttica RJ Rothschild BM DiDomenico LA. Chondrosarcoma of the proximal phalanx. J Foot Ankle Surg. (1999) 38:219–22. doi: 10.1016/S1067-2516(99)80056-8 10384362

[32] Hottya GA Steinbach LS Johnston JO van Kuijk C Genant HK. Chondrosarcoma of the foot: imaging, surgical and pathological correlation of three new cases. Skeletal Radiol. (1999) 28:153–8. doi: 10.1007/s002560050492 10231913

[33] Bovée JV van der Heul RO Taminiau AH Hogendoorn PC. Chondrosarcoma of the phalanx: A locally aggressive lesion with minimal metastatic potential: A report of 35 cases and a review of the literature. Cancer. (1999) 86:1724–32. doi: 10.1002/(SICI)1097-0142(19991101)86:9<1724::AID-CNCR14>3.0.CO;2-I 10547545

[34] Nakajima H Ushigome S Fukuda J. Case report 482: Chondrosarcoma (grade 1) arising from the right second toe in patient with multiple enchondromas. Skeletal Radiol. (1988) 17:289–92. doi: 10.1007/BF00401814 3212493

[35] Wiss DA. Chondrosarcoma of the first metatarsal. J Surg Oncol. (1983) 23:110–2. doi: 10.1002/jso.2930230213 6855239

[36] Wirbel RJ Remberger K. Conservative surgery for chondrosarcoma of the first metacarpal bone. Acta Orthop Belg. (1999) 65:226–9. 10427806

[37] Jakobson E Spjut HJ. Chondrosarcoma of the bones of the hand Report of 3 cases. Acta Radiol. (1960) 54:426–32. doi: 10.3109/00016926009171164 13789244

[38] Mulligan ME. How to diagnose enchondroma, bone infarct, and chondrosarcoma. Curr Probl Diagn Radiol. (2019) 48:262–73. doi: 10.1067/j.cpradiol.2018.04.002 29724496

[39] Behzadi AH Raza SI Carrino JA Kosmas C Gholamrezanezhad A Basques K. Applications of PET/CT and PET/MR imaging in primary bone Malignancies. PET Clin. (2018) 13:623–34. doi: 10.1016/j.cpet.2018.05.012 PMC7466825 30219192

[40] Bianchi S. Ultrasound and bone: a pictorial review. J Ultrasound. (2020) 23:227–57. doi: 10.1007/s40477-020-00477-4 PMC7441135 32419074

