# Supplementary figures and images for: Atypical cartilaginous tumor imaging findings in the distal phalanx of the left thumb: case report and literature review
Source: Front Oncol. 2025 Feb 24;15:1407012. doi: 10.3389/fonc.2025.1407012 (PMC11891022; doi:10.3389/fonc.2025.1407012)

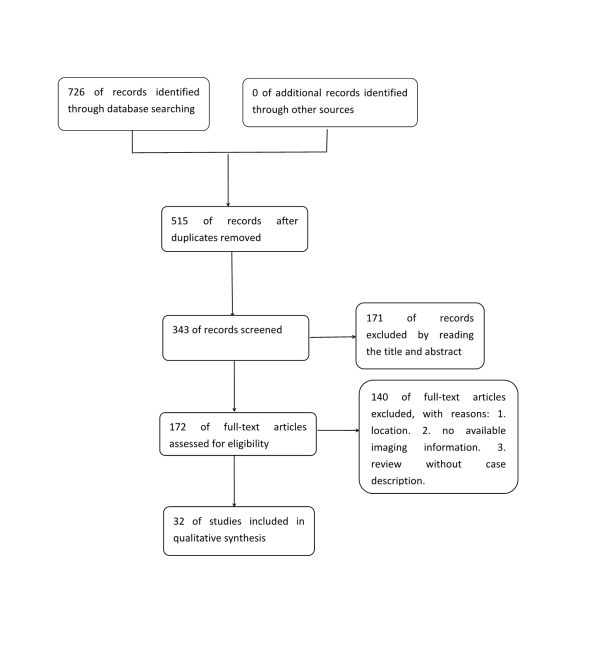

Supplement: Supplementary Figure 1 — A flowchart showing the literature search and analysis process. [file Image1.tif]
